# Supplementary material for: Heterogeneity in respiratory electron transfer and adaptive iron utilization in a bacterial biofilm
Source: Nat Commun. 2019 Aug 16;10:3702. doi: 10.1038/s41467-019-11681-0 (PMC6697725; doi:10.1038/s41467-019-11681-0)
Supplement: Supplementary file 1 — Supplementary Information [file 41467_2019_11681_MOESM1_ESM.pdf]

Supplementary Information by Qin et al

Title: "Heterogeneity in Respiratory Electron Transfer and Adaptive Iron Utilization in a Bacterial Biofilm"

1. **Supplementary Figures**
2. **Supplementary Tables**
3. **Supplementary References**

Supplementary Figures

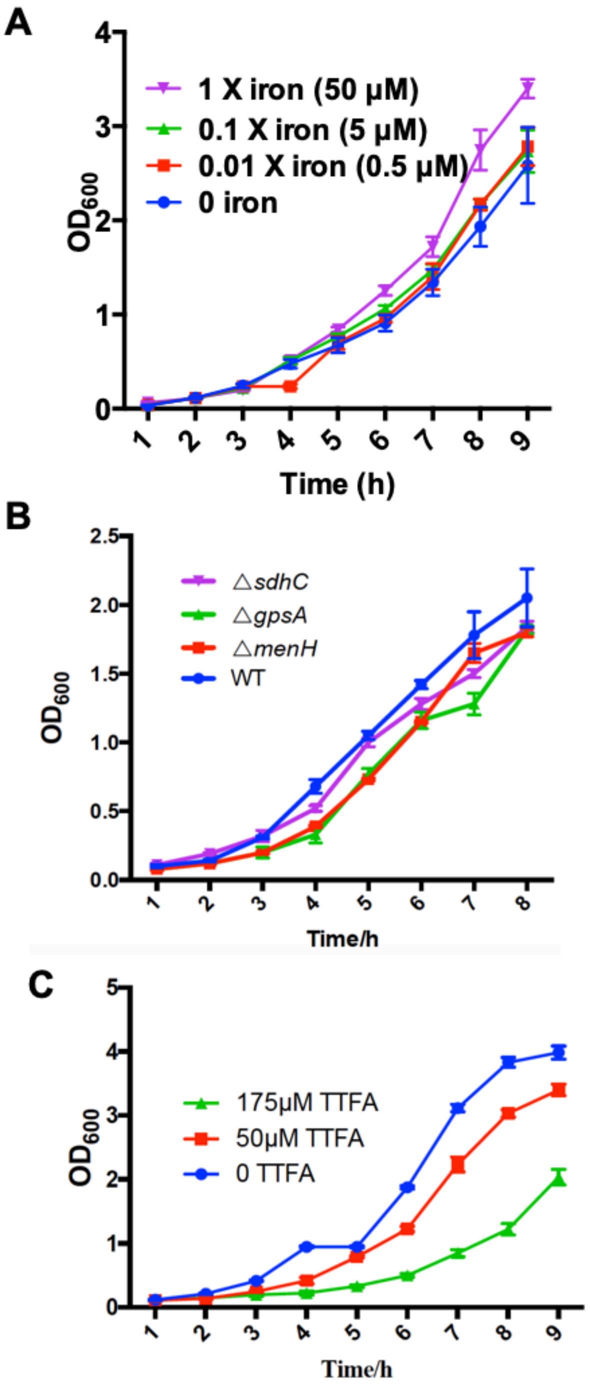

Supplementary Figure 1. Growth profiles of various *B. subtilis* strains under indicated conditions. (A) Growth profiles of *B. subtilis* 3610 under different media iron

conditions. Cells were inoculated in modified MSgg media supplemented with various amounts of $FeCl_3$ as indicated. Cells were grown at 37°C with shaking and cultural densities (OD_{600}) were periodically measured. **(B)** Growth curve of the Δ*sdhC*, Δ*menH* and Δ*gpsA* mutants in MSgg. Cells were grown in MSgg shaking cultures for a period of 8 hours at 37°C. Cells were collected every hour for the measurement of cell optical density (OD_{600}). Strains used in this experiment include 3610 (WT), Δ*sdhC*(YQ172), Δ*gpsA*(YQ219) and Δ*menH*(YQ218). **(C)** Growth curve of 3610 treated with different concentrations of TTFA. 3610 cells were inoculated in MSgg shaking cultures and grown at 37°C. TTFA was added at indicated concentrations at the beginning of the inoculation. Cells were collected every hour for the measurement of cell optical density(OD_{600}). No growth inhibition was observed when TTFA was added at the concentration of 50 μM, while a modest growth inhibition was observed at the concentration of 175 μM. The experiments were done in triplicate. Error bars represent standard deviations. Source data are provided as a source data file.

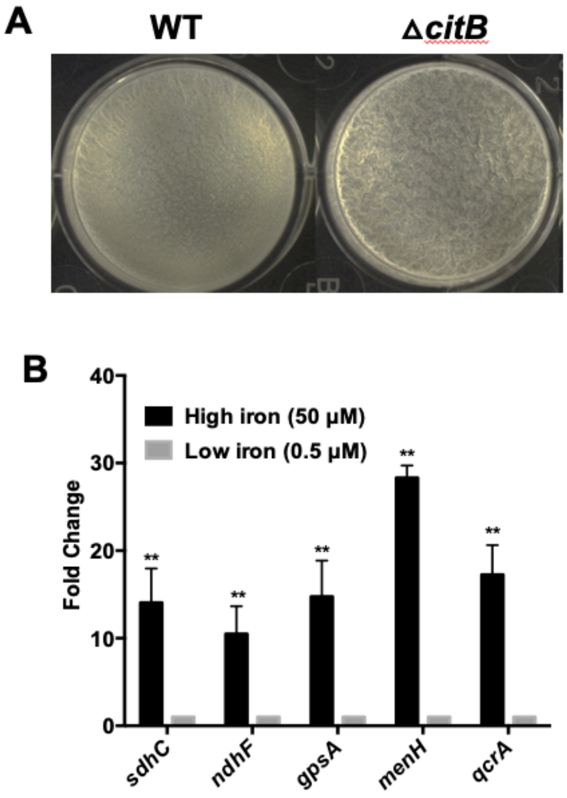

Supplementary Figure 2. (A) Biofilm formation by the WT and the Δ*citB* mutant. Cells were incubated in regular MSgg media for 48 hours at 30°C before the images were recorded. Strains used in this experiment include 3610 (WT) and Δ*citB*(YQ157). **(B)** Real-time PCR analyses to probe expression of the genes involved in electron transfer. Total RNAs were prepared from cells grown in MSgg in shaking but with two different ferric iron supplementations (50 μM vs 0.5 μM $FeCl_3$). Expression of *sdhC*, *ndhF*, *gpsA*, *menH*, and *qcrA* was found much higher in cells grown in media with 50 μM $FeCl_3$ than in media with 0.5 μM $FeCl_3$. The relative transcriptional level in the latter media was set at 1; fold changes represent the fold differences in transcription under two different media conditions. Statistical significance was assayed using unpaired *t-test* via Prism 6. Stars indicate P values < 0.01. Source data are provided as a source data file.

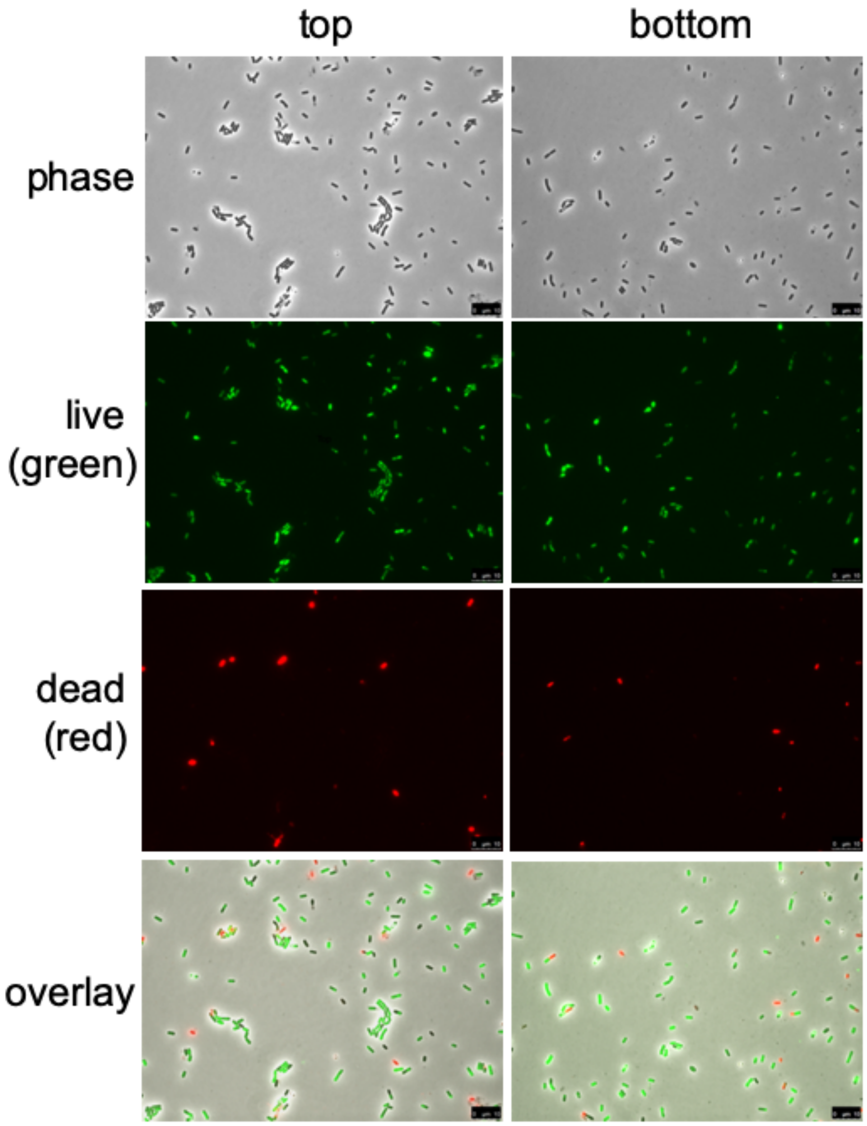

Supplementary Figure 3. Live/dead staining of 3610 cells collected from top and bottom layers of the pellicle biofilm. Top and bottom cells were collected as described in the methods. Dead cells were stained in red while live cells stained in green. Scale bars, 10 μm.

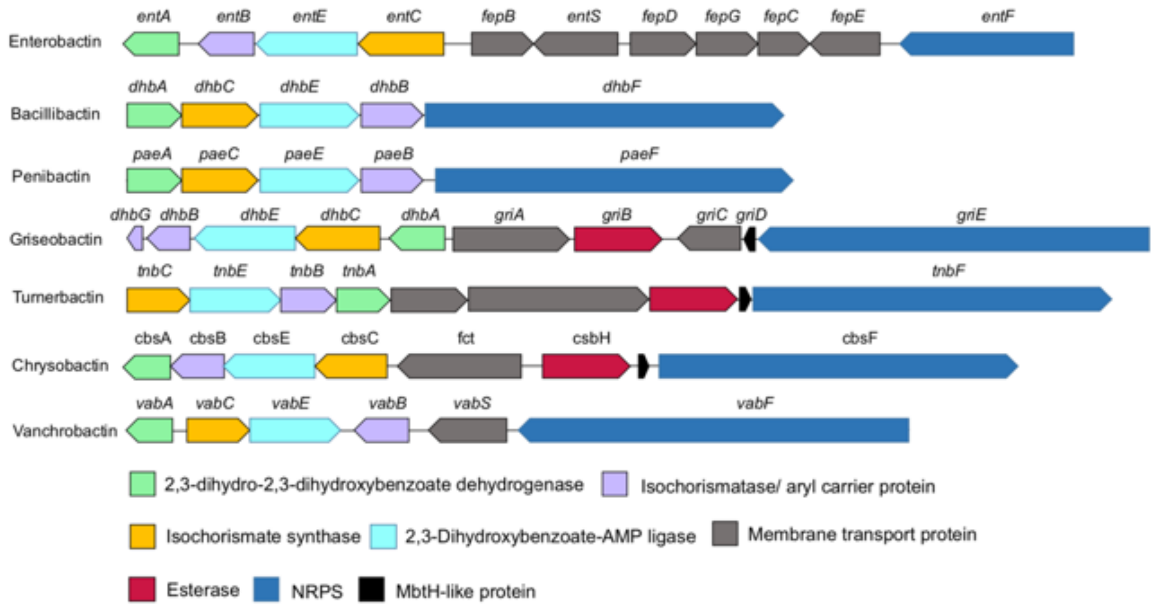

Supplementary Figure 4. Tricatechol siderophore biosynthetic gene clusters in bacteria. Shown are the homologous gene clusters involved in biosynthesis of bacillibactin and enterobactin-like tricatechol siderophores in various bacteria (modified from Reitz et al[1]). The *dbhF* homologous genes are not in the same operon with other biosynthetic genes except for the bacillibactin and penibactin pathways in the *Bacillus* spp.

Supplementary Tables

Supplementary Table 1: Strains and plasmids used in this study.

Strain	Parent	Genotype	Source
DH5α		An *E. coli* strain for molecular cloning	Invitrogen
PY79		A laboratory strain of *B. subtilis* for genetic transformation	[2]
168		A domesticated strain of *B. subtilis*	[3]
NCIB3610		An undomesticated strain of *B. subtilis* capable of biofilm formation	[4]
BKE32000	168	Δ*dhbA*::*erm*	BGSC
BKE31970	168	Δ*dhbB*::*erm*	BGSC
BKE31990	168	Δ*dhbC*::*erm*	BGSC
BKE31980	168	Δ*dhbE*::*erm*	BGSC
BKE31960	168	Δ*dhbF*::*erm*	BGSC
BKE18000	168	Δ*citB*::*erm*	BGSC
BKE18450	168	Δ*gltA*::*erm*	BGSC
BKE28450	168	Δ*sdhC*::*erm*	BGSC
BKE22560	168	Δ*qcrA*::*erm*	BGSC
BKE22750	168	Δ*menH*::*erm*	BGSC
BKE22830	168	Δ*gpsA*::*erm*	BGSC
BKE01830	168	Δ*ndhF*::*erm*	BGSC
YQ90	3610	Δ*dhbA*::*erm*	This study
YQ91	3610	Δ*dhbB*::*erm*	This study
YQ92	3610	Δ*dhbC*::*erm*	This study
YQ93	3610	Δ*dhbE*::*erm*	This study
YQ94	3610	Δ*dhbF*::*erm*	This study
YQ97	YQ90	Δ*dhbA, maker-less deletion*	This study
YQ98	YQ91	Δ*dhbB, maker-less deletion*	This study
YQ99	YQ92	Δ*dhbC, maker-less deletion*	This study
YQ100	YQ93	Δ*dhbE, maker-less deletion*	This study
YQ101	YQ94	Δ*dhbF, maker-less deletion*	This study
YQ120	YQ97	Δ*dhbA, maker-less deletion,* Δ*fur::erm*	This study
YQ138	PY79	*amyE*::P_{dhb}-*lacZ::spec*^{R}	This study
YQ141	3610	*amyE*::P_{dhb}-*lacZ::spec*^{R}	This study
YQ142	YQ141	Δ*fur*::*erm, amyE*::P_{dhb}-*lacZ*::*spec*^{R}	This study
YQ157	3610	Δ*citB*::*erm*	This study
YQ170	3610	Δ*gltA*::*erm*	This study
YQ172	3610	Δ*sdhC*::*erm*	This study
YQ191	YQ172	Δ*sdhC*::*erm, sacA*::P_{tapA}-*mKate2, kan*^{R}	This study
YQ218	3610	Δ*menH*::*erm*	This study

YQ219	3610	Δ*gpsA*::*erm*	This study
YQ220	3610	Δ*qcrA*::*erm*	This study
YQ221	3610	Δ*ndhF*::*erm*	This study
YQ255	YQ141	Δ*abrB*::*erm, amyE*::P_{dhb}-*lacZ*::$spec^R$	This study
KG168	3610	Δ*fur*::*erm*	Lab Stock
TMN503	3610	*sacA*::P_{tapA}-*mKate2, kan*^{R}	5
YC141	3610	Δ*lutABC*::$spec^R$	6
YC668	3610	Δ*abrB::kan*^{R}	7
CY167	3610	Δ*pks*::tet^R	Lab Stock
CY168	3610	Δ*pps*::chl^R	Lab Stock
**Plasmid**			
pDG1728		An *amyE* integration vector with a promoter-less lacZ, $spec^R$, amp^R	8
pDR244		Loop-out plasmid for generating markerless deletions in the *Bacillus subtilis* BKE knockout collection	BGSC

Abbreviations for the antibiotic resistance genes: amp, ampicillin; chl, chloramphenicol; erm, erythromycin; kan, kanamycin; spec, spectinomycin; tet, tetracycline.

Supplementary Table 2: Oligonucleotides used in this study.

Primers (5'>3')	
P_{dhb}-F	GTACGAATTCGACGGACCGCATCTATCAAT
P_{dhb}-R	GTACAAGCTTTCGCTCTAAATGCCAAACAG
sdhC_qPCR-F	GATAGCCTGCCTTTCAGGTATG
sdhC_qPCR-R	GCTGTATTGACCGGCGTTAT
ndh_qPCR-F	GGCGTACAAGGTAACCCATTAG
ndh_qPCR-R	CTGTCTCCAGCAACGAATACA
gpsA_qPCR-F	GGTTTAGGGTACGGTGACAAT
gpsA_qPCR-F	CCAGAGAACGTCAAGGGATTT
menH_qPCR-F	ATGCGATGGAGCTTCCTTT
menH_qPCR-R	GCTTCACTACACGTCTCATCTC
qcrA_qPCR-F	GATGCCTGGTATGAGTCAGAAG
qcrA_qPCR-R	CAGTTCACCGTACACCCTAAAT
dhbA_ qPCR -F	CGAAGATCGGGTTCGATTGTA
dhbA_ qPCR -R	GGCCAAGGCATTTCGTAAAC
dhbB_ qPCR -F	TGGAGATACAGCGCGTTTAAG
dhbB_ qPCR -R	CAGCCGATATGGGCGTAAAT
dhbC_ qPCR -F	AGGTCGTTTCCAACCCATTAG
dhbC_ qPCR -R	TGAAGATCCTTTGCGGAAGAA
dhbE_ qPCR -F	GCAGAGGGTCTCGTCAATTATAC
dhbE_ qPCR -R	GATCATCCCAAACACGCATTTC
dhbF_ qPCR -F1	CTTCAATATTCTTTGACCGG
dhbF_ qPCR -R1	ACATGCAGCGATTCCGCTTC
dhbF_ qPCR -F2	GAACTTATACGGTCCCACTG
dhbF_ qPCR -R2	CAATATAAAGCTCTCCGACA
dhbF_ qPCR -F3	TACAGAAACGACGGTGCATG
dhbF_ qPCR -R3	CTCCTGATACATACAGCTCT

Supplementary References

1 Reitz, Z. L., Sandy, M. & Butler, A. Biosynthetic considerations of triscatechol siderophores framed on serine and threonine macrolactone scaffolds. *Metallomics* **9**, 824-839, doi:10.1039/c7mt00111h (2017).

2 Schroeder, J. W. & Simmons, L. A. Complete Genome Sequence of *Bacillus subtilis* Strain PY79. *Genome Announc* **1**, e01085-01013, doi:10.1128/genomeA.01085-13 (2013).

3 Harwood, C. R. & Wipat, A. Sequencing and functional analysis of the genome of *Bacillus subtilis* strain 168. *Febs Lett* **389**, 84-87, doi:Doi 10.1016/0014-5793(96)00524-8 (1996).

4 Branda, S. S., Gonzalez-Pastor, J. E., Ben-Yehuda, S., Losick, R. & Kolter, R. Fruiting body formation by Bacillus subtilis. *P Natl Acad Sci USA* **98**, 11621-11626, doi:DOI 10.1073/pnas.191384198 (2001).

5 Norman, T. M., Lord, N. D., Paulsson, J. & Losick, R. Memory and modularity in cell-fate decision making. *Nature* **503**, 481-486, doi:10.1038/nature12804 (2013).

6 Chai, Y. R., Kolter, R. & Losick, R. A Widely Conserved Gene Cluster Required for Lactate Utilization in Bacillus subtilis and Its Involvement in Biofilm Formation. *J Bacteriol* **191**, 2423-2430, doi:10.1128/Jb.01464-08 (2009).

7 Shemesh, M. & Chai, Y. R. A Combination of Glycerol and Manganese Promotes Biofilm Formation in *Bacillus subtilis* via Histidine Kinase KinD Signaling. *Journal of Bacteriology* **195**, 2747-2754, doi:10.1128/Jb.00028-13 (2013).

8 Anne-Marie Gurout-Fleury, N. F., Patrick Stragier. Plasmids for ectopic integration in Bacillus subtilis. *Gene* **180**, 57-61 (1996).
